# Cancer Patterns and Barriers to Care Among Socioeconomically Vulnerable Populations in Tripoli: A Descriptive Study from a Local NGO

**DOI:** 10.3390/diseases14050170

**Published:** 2026-05-12

**Authors:** Mouhamad J. Darwich, Dalal Ksair, Zein Adra, Rafaela-Yomn Naji, Bushra Sayed, Rihab Nasr, Zeina Dassouki

**Affiliations:** 1 Sanabel Nour, Research and Development Unit, Medical Department, Tripoli 1300, Lebanon; mhd.30nov.1991@gmail.com (M.J.D.); kasirdalal@gmail.com (D.K.); zeinadra@gmail.com (Z.A.); rafaela-naji@live.com (R.-Y.N.); bouchrasayed0@gmail.com (B.S.); 2 Department of Anatomy, Cell Biology and Physiological Sciences, Faculty of Medicine, American University of Beirut, Beirut P.O. Box 11-0236, Lebanon; 3 Department of Medical Laboratory Sciences, Faculty of Health Sciences, University of Balamand, Beirut 55251, Lebanon

**Keywords:** cancer epidemiology, colorectal cancer, breast cancer, Lebanon, socioeconomic disparities, screening, access to care, health equity

## Abstract

Background/Objectives: Cancer patterns in low-resource and crisis-affected settings are poorly characterized, particularly among socioeconomically vulnerable populations. This study aimed to describe cancer distribution, age at diagnosis, and barriers to care among patients presenting to a non-governmental organization (NGO) in Tripoli, Lebanon. Methods: We conducted a retrospective analysis of patients with histopathologically confirmed cancers presenting to a single NGO. Sociodemographic, clinical, and behavioral data were extracted from medical records. Socioeconomic status (SES) was assessed using a validated composite scale. Age-standardized proportions (ASPs) were calculated using GLOBOCAN and WHO standard weights. Barriers to care were categorized into financial, geographic, system-level, and sociocultural domains. Associations were assessed using chi-square tests and regression models. Results: Breast cancer was the most common malignancy (32.0%), followed by colorectal (CRC: 9.8%). A total of 440 patients were included. Colorectal cancer (CRC) was the second-most common malignancy, with 37% of cases occurring before age 50. Breast cancer accounted for nearly half of female cancers. Smoking-related malignancies, particularly bladder and lung cancers, were prominent. Sex differences were cancer-specific, with male sex associated with bladder cancer but not overall cancer distribution. Barriers to care were highly prevalent: 97.3% reported at least one financial barrier, 95.4% system-level barriers, and 72.4% geographic barriers. Low SES was significantly associated with geographic barriers (*p* < 0.001). Conclusions: Cancer patterns in this vulnerable population are characterized by early-onset disease, a high burden of smoking-related cancers, and pervasive barriers to care. These findings highlight the importance of integrating SES and access-related variables into cancer surveillance systems and support the development of targeted, equity-focused interventions.

## 1. Introduction

Cancer is a leading cause of death worldwide, with an estimated 19.3 million new cases and nearly 10 million cancer-related deaths in 2020 [1]. Low- and middle-income countries (LMICs) bear a disproportionate share of the global cancer burden; they account for 70% of cancer-related deaths, largely due to late-stage diagnoses and limited access to treatment, deepened by sociocultural, geographic, and financial barriers [2].

Lebanon, reclassified by the World Bank in 2022 as an LMIC, currently has the highest cancer incidence in the Arab world [3,4]. The country’s fragile healthcare system is expected to face further strain as cancer cases are projected to rise by approximately 60% over the next decade, reaching an estimated 20,000 new cases by 2035 [5]. This increase is likely associated with population aging, high smoking rates, westernized diets, sedentary lifestyles, and air pollution [4,6]. Most cancers in Lebanon are diagnosed at late stages, leading to low survival rates [7].

Since 2019, Lebanon’s economic collapse, regional instability, and the Beirut port explosion have devastated the healthcare system [5,8]. These events have led to an increase in transportation costs, a lack of health insurance, and increased prices and shortages in cancer medications due to subsidy cuts [9]. These factors may contribute to a higher likelihood of late-stage cancer diagnoses [1].

Socioeconomic disparities have further deepened, exacerbating inequities in cancer care access [10]. A study revealed that national mammography campaigns have failed to effectively reach disadvantaged areas like Tripoli in the north, where significant knowledge gaps regarding screening persist among lower socioeconomic groups [11]. Despite increased mammography use, advanced-stage diagnoses have risen. This reflects persistent information and access inequities, as most resources remain concentrated in the capital, Beirut [10]. Women from higher SES backgrounds, particularly in Beirut, are significantly more likely to undergo breast cancer screening, while socioeconomically vulnerable women are overrepresented among those unaware of mammography [11].

Beyond screening, cancer patients from vulnerable communities face multiple barriers to timely care, with financial costs being the most significant [12,13]. The economic collapse may have exacerbated these barriers, along with persistent misconceptions about cancer and the bureaucracy involved in accessing anti-cancer medications [14,15]. These communities often lack health insurance and must rely on out-of-pocket payments within Lebanon’s highly privatized and fragmented healthcare system [14].

Efforts to address these challenges are further complicated by major gaps in cancer data. The Lebanese Ministry of Public Health established the National Cancer Registry (NCR) in 1998 to track cancer incidence by age, sex, time, and primary site [5]. However, the registry was last updated in 2017, leaving out critical information on cancer trends following the country’s recent crises. Moreover, the NCR lacks data on the geographical distribution of cases and the SES of patients, hindering a full understanding of the profile of cancer patients in vulnerable regions and communities like Tripoli.

In light of these gaps, we aimed to profile cancer cases among vulnerable populations in Tripoli and identify barriers to cancer healthcare. Such data will help guide tailored, culturally sensitive prevention and care services to reduce Lebanon’s growing cancer burden.

## 2. Materials and Methods

### 2.1. Setting

The study was conducted at Sanabel Nour, a local non-governmental organization (NGO) based in Tripoli, North Lebanon. The local NGO has a significant reach and provides medical and social assistance to cancer patients from underserved communities in Tripoli and its outskirts.

### 2.2. Study Design

This study employed a retrospective chart review design, utilizing cancer patient records from an NGO-based setting in North Lebanon. Sanabel Nour routinely collects and records demographic, SES, and medical data as part of its standard beneficiary assessment process. This assessment is followed by detailed medical questions. This study is based on patients presenting to a single NGO and therefore represents a highly selected population experiencing barriers to care. As such, findings are not in-tended to be representative of the general population of Tripoli or Lebanon. The study is subject to potential selection bias, as it includes only patients who sought care at this NGO, and to information bias due to reliance on retrospective medical records. These factors are further addressed in the limitations.

### 2.3. Process

After obtaining approval from the University of Balamand Institutional Review Board (IRB), the senior researcher screened the health database at Sanabel Nour using the keywords “malignancy”, “tumor”, and “cancer” to identify all patients with these conditions who presented to the NGO between January 2022 and July 2024. Each patient was assigned a randomly generated code to ensure de-identification. Trained research assistants then reviewed each de-identified medical record to extract the information outlined below. The extracted data were entered and analyzed using IBM SPSS version 27.

### 2.4. Data Collected

The following data were extracted:

Demographic information: sex, residence, date of birth, and nationality. All included patients were residents of Tripoli. Residency was defined based on the patient’s place of residence at the time of presentation to the NGO, as recorded in the medical and social assessment records. The district of Tripoli was subdivided into 11 areas (Abou Samra, Beb El Ramel, Beddawi, Mina, New Tripoli, Old Tripoli, Qalmoun, Qoubbeh, Tabbaneh, West Tripoli, Zehriyeh).

Two age variables were derived: age at first cancer diagnosis and age at presentation to the NGO. Age at diagnosis was obtained from histopathology reports and used for all cancer-specific analyses (e.g., age distribution and early-onset classifications), whereas age at NGO presentation was used for descriptive and sociodemographic analyses where relevant.

Socioeconomic status (SES) was assessed using the Socioeconomic Status Composite Scale (SES-C) [16]. The SES-C is a multidimensional tool developed and validated in Lebanon (2022). The scale integrates ten indicators, including education, employment, income, perceived social class, financial well-being, and household crowding. Scores range from 6 to 28 and are categorized into four levels (very low to high). The SES-C has demonstrated good construct validity and excellent internal consistency (Cronbach’s α = 0.914) in the Lebanese context. Convergent validity was demonstrated through significant correlations between the composite score and its components, particularly income, perceived social class, and financial well-being (all *p* < 0.001). Additionally, higher SES-C scores were significantly associated with favorable health indicators, including better access to healthcare and a lower burden of chronic disease, supporting its criterion-related validity in the Lebanese context. Based on the score, patients’ SES is categorized as very bad, bad, average, or good.

Tumor information: primary site, pathological diagnosis, age at diagnosis, local lymph node involvement at diagnosis, metastasis at diagnosis, and metastasis at presentation to the NGO. Future manuscripts will address site-specific analyses.

Data collection form was aligned as closely as possible with the NCR structure [17], including the classification of primary site and age groups. The data collection form is provided in the Supplementary Material. The National Cancer Registry (NCR) data collection form is publicly available through the Lebanese Ministry of Public Health [17]. Cancer types were categorized according to ICD codes (C00–C97). As indicated by Bray et al. [18], we combined colon, rectum, and anus cancers as colorectal cancer (C18–C21). All lymphomas were grouped into one group (lymphoma), which was further divided into Hodgkin disease (C81) and non-Hodgkin lymphoma, and all leukemias were combined into one group (leukemia), which was further divided into lymphoid leukemia (C91) and myeloid leukemias (C92, C93, and C94).

Finally, information about smoking was included due to its established association with specific malignancies (e.g., lung and bladder cancers), while SES and access-related variables were included to examine structural determinants of delayed diagnosis and barriers across the care continuum.

Barriers were operationalized as eleven yes/no questionsand were divided into four major domains: Financial barriers refer to direct or indirect economic constraints that limit access to cancer care, including the inability to afford medications, treatment costs, or associated expenses. Geographic barriers refer to physical or logistical challenges related to distance, transportation, or access to healthcare facilities. System-level barriers refer to structural issues within the healthcare system, including the availability of services, care coordination, communication with providers, and timeliness of care delivery. Social and cultural barriers refer to interpersonal and sociocultural factors that influence care-seeking behavior and support systems, including stigma, health literacy, and the availability of social or emotional support.

### 2.5. Inclusion and Exclusion Criteria

All cancer patients who presented to the NGO from January 2022 until July 2024 were considered eligible. The inclusion criteria were being a resident of Tripoli, having an SES assessment below good (very bad, bad, or average), and having a medically proven malignancy. Only patients with diagnoses confirmed with a histopathological report were included.

A total of 712 patient records were initially identified from the Sanabel Nour medical database based on the keywords “malignancy”, “tumor”, and “cancer” between January 2022 and July 2024. After applying the exclusion criteria, 283 patients were excluded from the final analysis for the following reasons: (1) benign tumors (*n* = 37), (2) lack of proof or insufficient documentation of malignancy (*n* = 116), (3) non-residence in Tripoli (*n* = 107), and (4) an SES score of “good” (*n* = 23).

### 2.6. Baseline Sample Characteristics

The final sample size consisted of 440 patients. The majority of the cohort was female (*n* = 288; 65.6%). Geographically, the largest proportion of patients resided in Qoubbeh (121 patients, 27.5%). Patients were, on average, 53 ± 15.31 years old at the time of presentation to the NGO.

### 2.7. Ethical Considerations

All incoming patients to Sanabel are informed that their medical information may be used for research purposes, with a clear option to opt out. If a patient or caregiver refuses, this is noted in their database file, and their data will not be included in any study. All medical files were de-identified and assigned a code prior to data extraction. The research team received training in data extraction protocols. This research was approved by the University of Balamand Institutional Review Board (IRB).

### 2.8. Statistical Analysis

Descriptive statistics were calculated, including frequencies and percentages, for each cancer type according to the National Cancer Registry (NCR) classification. Data were further stratified by age and sex. Scale variables were summarized using means and standard deviations, while categorical variables were described using frequencies and percentages. Missing data were handled using listwise deletion for analyses where variables were incomplete, and for categorical variables, valid percentages were reported to account for missing responses.

Age-standardized proportions (ASPs) were calculated using a direct standardization approach to adjust for differences in age structure. Because the dataset is not population-based and does not include a defined denominator representing the population at risk, true incidence or prevalence rates could not be computed.

Instead, the observed distribution of cancer cases within the study sample was standardized using age distributions from GLOBOCAN 2022 estimates for Lebanon and weighted according to the World Health Organization (WHO) standard population [19]. These standardized measures reflect relative patterns in the age distribution of cases and are presented for descriptive comparison purposes only. They should not be interpreted as population-level incidence or prevalence rates.

Depending on data distribution and variable type, chi-square tests, *t*-tests, and non-parametric equivalents (Wilcoxon or Fisher’s exact tests) were used to compare observed and national values. Unless otherwise specified, differences between observed and expected proportions were tested using chi-square or Fisher’s exact tests. Sex differences in the distribution of cancer types were assessed using two approaches based on case counts per cancer site. For cancer types with ≥10 cases, a binary logistic regression model was performed, with sex as the independent variable (male as the reference category) and each cancer type entered as a binary dependent variable (presence vs. absence). Model fit was evaluated using the omnibus chi-square test of model coefficients, and effect size was reported using odds ratios (ORs) with 95% confidence intervals (CIs). Cox & Snell and Nagelkerke R^2^ values were also calculated. For cancer types with <10 cases, Fisher’s exact test was used to assess the association between sex and cancer type due to small cell counts. Corresponding odds ratios and 95% CIs were calculated. Sex-specific cancers (breast, cervical, ovarian, uterine, and prostate) and cases categorized as “other” were excluded from inferential analyses comparing sex differences. Additionally, a multinomial logistic regression model was conducted, including major non-sex-specific cancer types to assess the association between sex and cancer type simultaneously. Given sample size limitations, results were interpreted cautiously. Finally, associations between smoking status and cancer type were assessed using chi-square or Fisher’s exact tests, with odds ratios calculated where appropriate. Findings from subgroups with small sample sizes should be interpreted as exploratory.

Normality was assessed by examining Q-Q plots and histograms. Boxplots were used to check for outliers. The asymptotic significance for all tests was two-tailed and set at a *p*-value of 5%.

## 3. Results

### 3.1. The Most Common Types of Cancer

Our sample consisted of 440 cancer patients. The five most common cancers were breast (32.0%), colorectal (CRC: 9.8%), bladder (8.4%), lymphoma (7.5%), and lung (7.0%). When the patients were segregated by sex, the five most frequent types of cancer in females were, in decreasing order: breast, colorectal, lymphoma, lung cancer, and bladder and uterine cancers, which tied for the fifth rank. For male patients, the five most frequent types of cancer were, in decreasing order: bladder, colorectal, prostate, lymphoma, and lung. A detailed summary of these findings is provided in Table 1.

Age-standardized proportions (ASPs) were standardized using GLOBOCAN 2022 population age structures for Lebanon due to the lack of a defined population at risk in Tripoli, Lebanon, and weighted using the standard population of the World Health Organization [20]. Age-standardized proportions (ASPs) were calculated to account for differences in age structure and to allow comparison of relative case distributions. The highest age-standardized proportion was observed for breast cancer (ASP = 5.05), followed by colorectal (0.78) and bladder (0.73). These standardized proportions reflect the relative distribution of cancer cases across age groups within the study population. They do not represent incidence rates and should be interpreted as descriptive measures rather than population-level estimates.

### 3.2. Sex Distribution Across Cancer Types

Among cancer types with ≥10 cases per type, sex was significantly associated with colorectal and bladder cancers; CRC showed a modest but statistically significant association with sex (13.8% of male patients had CRC vs. 7.6% of female patients, χ^2^ = 4.126, *p* = 0.042), with higher odds in males compared to females (OR = 1.94, 95% CI: 1.03–3.65). Meanwhile, bladder cancer demonstrated a strong association (17.1% of male patients had bladder cancer vs. 3.8% of female patients, χ^2^ = 21.51, *p* < 0.001), with markedly higher odds in males (OR = 5.20, 95% CI: 2.49–10.85). No statistically significant sex differences were observed for lymphoma, lung cancer, or leukemia. Laryngeal cancer showed a borderline association (*p* = 0.088), with a trend toward higher odds in males (4.6% of male patients had laryngeal cancer vs. 1.7% of female patients, OR = 2.73, 95% CI: 0.85–8.76), though this did not reach statistical significance. The explained variance for all models was low (Nagelkerke R^2^ ranging from 0.002 to 0.109), indicating that sex alone accounted for a small proportion of variability in cancer occurrence.

For cancer types with <10 cases, Fisher’s exact test was used, and results were interpreted with caution. Stomach cancer showed a significant association (5.3% of male patients had stomach cancer vs. 0.3% of female patients, *p* = 0.001), with lower odds in females compared to males (OR = 0.063, 95% CI: 0.008–0.506). Kidney cancer was also significantly associated with sex (*p* = 0.023), again indicating lower odds in females (3.9% of male patients had kidney cancer vs. 0.7% of female patients, OR = 0.17, 95% CI: 0.034–0.854). No significant sex differences were found for multiple myeloma, brain and CNS tumors, connective and soft tissue tumors (*p* = 0.241), or thyroid cancer. The heterogeneous category “Other Sites” was excluded from this step of the analysis.

A multinomial logistic regression model was performed, including non-sex-specific cancers with more than 10 cases. The overall model was not statistically significant (χ^2^(5) = 9.57, *p* = 0.088), suggesting that sex did not significantly predict cancer type when analyzed simultaneously. Parameter estimates indicated a higher likelihood of bladder cancer relative to CRC among males (OR = 2.48, 95% CI [0.98–6.24], *p* = 0.055), although this did not reach statistical significance. No significant differences were observed for lymphoma, lung cancer, leukemia, or laryngeal cancer. These findings are consistent with the stratified analyses, which identified a strong association between male sex and bladder cancer, although this effect was attenuated when analyzed within a multinomial framework. A detailed breakdown of sex distribution per cancer type and the multinomial model can be found in the Annex (Tables S1–S4).

### 3.3. Age at Diagnosis of the Most Common Cancer Types

**Figure 1:**
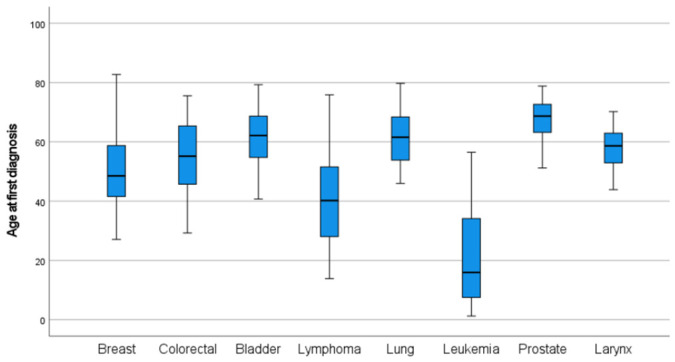
Boxplots of the age distribution at diagnosis of the most common cancer types.

**Figure 2:**
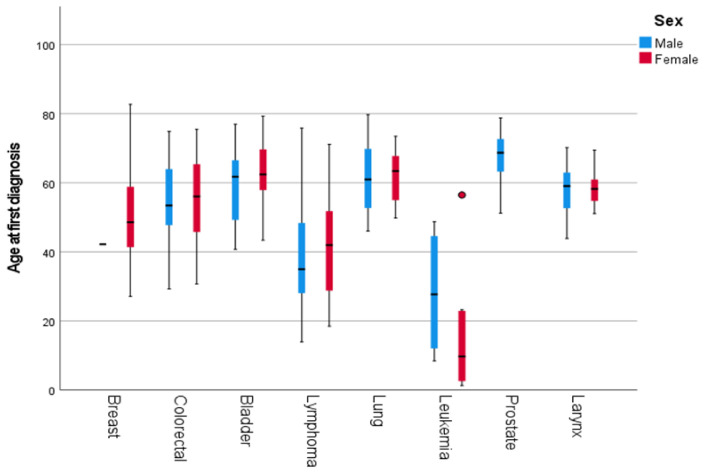
Boxplots illustrating the distribution of the age at first diagnosis for the most common types of cancer, stratified by sex. The horizontal line in the male breast cancer group represents a single patient (*n* = 1). Dots indicate outliers (values beyond 1.5 × IQR).

Age at diagnosis varied substantially across cancer types. Cancers such as lung, bladder, colorectal, and prostate were predominantly diagnosed at older ages, with 93.3%, 75.7%, 62.8%, and 100% of cases occurring in individuals aged ≥50 years, respectively. In contrast, malignancies such as lymphoma, brain and central nervous system tumors, and leukemia were observed at younger ages, with mean ages at diagnosis of 40.85, 37.63, and 21.34 years, respectively. Breast cancer showed an intermediate pattern, with 44% of cases occurring in individuals aged ≥50 years. Detailed mean and median age at diagnosis, along with the proportion of patients diagnosed at ≥50 and ≥60 years by cancer type, are presented in Table S5 of the Supplementary Material. We calculated the mean age at diagnosis for each major cancer type, stratified by sex. For female patients, the mean age at diagnosis ranged from 42.6 years (lymphoma) to 63.6 years (bladder cancer). As for male patients, the mean age at diagnosis ranged from 28 years (lymphoma) to 67 years (prostate cancer). The distribution of the age at diagnosis of the most common types of cancer is illustrated in Figure 1 and is further stratified by sex in Figure 2.

Age stratification by sex was omitted for cancer types with ≤10 cases per group. These included leukemia (21.34 ± 18.21 years), laryngeal cancer (58.23 ± 7.79), stomach cancer (55.99 ± 13.50), multiple myeloma (58.72 ± 10.63), kidney carcinoma (49.89 ± 7.87), brain and CNS cancers (37.63 ± 24.68), and sarcomas (40.98 ± SD = 18.16). For all cancer patients, regardless of primary site and sex, the average age at diagnosis was found to be 50.69 ± 15.93 years. These results are detailed in Table 2. Independent-samples *t*-tests showed no significant sex differences in age at diagnosis across all cancer sites (*p* > 0.05).

### 3.4. Patients with Colorectal Cancer (CRC)

CRC affected male and female patients in our sample nearly equally (*n* = 21 males and *n* = 22 females). The male-to-female (M/F) ratio was 0.95. This ratio did not differ significantly from the national ratio of 1.14 (χ^2^(1, *N* = 43) = 0.344, *p* = 0.56).

**Figure 3:**
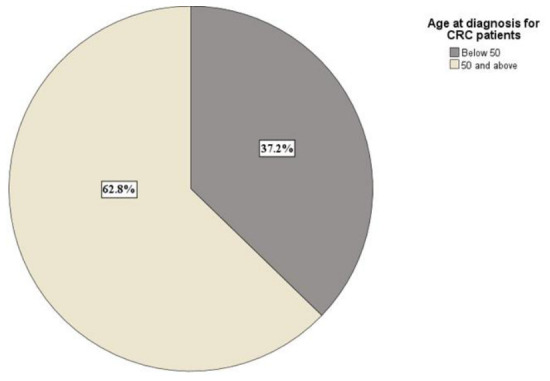
Percentage distribution of patients with early-onset (<50 years) vs. older-onset (≥50 years) CRC.

Early-onset colorectal cancer (EOCRC) is defined as a CRC diagnosis below the age of 50 years [21]. In our sample, 37.26% (*n* = 16) of patients with CRC had EOCRC (Figure 3). We compared the proportion of early-onset (<50 years) versus older-onset (≥50 years) CRC cases; the difference was not significant (χ^2^(1, *N* = 43) = 2.814, *p* = 0.09). We examined whether age group (<50 vs. ≥50 years) was associated with sex among CRC patients; no significant association was found ((χ^2^(1) = 0.014, *p* = 0.91).

The Lebanese national mean age of CRC diagnosis is 66 years for men and 65 years for women, according to the Lebanese Ministry of Public Health (MoPH) 2019 report [22].

In our sample, we found that male patients were diagnosed at a significantly younger age than the national mean. The mean difference was 11.49 years, t(20) = −4.07, *p* < 0.001, 95% CI [5.60, 17.38], indicating a large effect size, d = 0.85. Similarly, female patients were diagnosed at a significantly younger age than the national mean, with a mean difference of 9.98 years, t(21) = −3.25, *p* = 0.004, 95% CI [3.59, 16.36], representing a moderate to large effect size, d = 0.67 (Table 3). These findings highlight that the age of CRC diagnosis in our sample is markedly lower than the national average for both sexes.

### 3.5. Patients with Breast Cancer

In Lebanon, breast cancer accounts for approximately 38.6% of all female cancers [23]. In our sample of 288 female cancer patients, 140 (48.6%) were diagnosed with breast cancer. We compared the proportion of breast cancer cases in our sample (48.6%) with the national figure (38.6%), finding a significantly higher proportion (χ^2^ = 12.33, *p* = 0.0004, Cohen’s w = 0.21, indicating a small-to-moderate effect size.).

The Lebanese national median age for breast cancer diagnosis is 49.8 years [24]. In our sample, the median age at diagnosis for female breast cancer patients was slightly lower, at 48.57 years. The median age at diagnosis in the sample (48.6 years) did not differ significantly from the national median (49.8 years; Z = −0.30; *p* = 0.76; *N* = 141).

Note that, for both breast cancer and CRC, comparisons with national age at diagnosis were based on crude means and did not account for differences in population structure.

### 3.6. Bladder Cancer and Prostate Cancer

To assess the representativeness of the sample, we compared the observed frequencies for bladder and prostate cancers to national data.

First, the sex distribution of bladder cancer cases in the sample (*N* = 37) was compared to the national male-to-female ratio of approximately 4.3:1 (81.1% male, 18.9% female) as reported by the Lebanese NCR and corroborated by Lakkis et al. [25]. The observed distribution (70.3% male, 29.7% female) did not differ significantly from national proportions χ^2^(*N* = 37) = 2.82, *p* = 0.09.

Similarly, the proportion of bladder cancer among male cancer patients (26 of 152; 17.1%) was compared to the national estimate of 18.5% [24]. This difference was also not statistically significant, χ^2^(*N* = 152) = 0.20, *p* = 0.66.

Finally, prostate cancer prevalence among male patients (9.9%; 15 of 152) was tested against a projected national proportion of 14.2% [26]. No significant difference was observed, χ^2^(*N* = 152) = 2.60, *p* = 0.11.

Taken together, these results suggest that the sample does not significantly deviate from national distributions in terms of sex ratio for bladder cancer or the proportions of bladder and prostate cancer among male patients.

### 3.7. Lung Cancer

Sex distribution among lung cancer patients (12 males, 18 females) showed no significant difference, χ^2^ = 1.20, *p* = 0.27, indicating an approximately equal sex distribution within the sample. However, this distribution significantly deviated from national lung cancer incidence trends in Lebanon, where the male-to-female ratio is approximately 2.24:1 [27] (expected counts: 21 males, 9 females), χ^2^ = 12.86, *p* < 0.001, indicating an overrepresentation of females in the sample. Lung cancer patients were significantly more likely to be smokers than non-smokers (22 smokers vs. 7 non-smokers), χ^2^ = 9.14, *p* = 0.002. Smoking was prevalent in both sexes, with 7 out of 12 males and 15 out of 17 females identified as smokers (one female patient was excluded due to missing smoking status data). No significant association was found between sex and smoking status, though the trend approached significance, χ^2^ = 3.43, *p* = 0.06, suggesting a potential trend toward higher smoking rates among female patients.

Lung cancer patients were categorized into six pathological subtypes: adenocarcinoma (*n* = 12), squamous cell carcinoma (*n* = 8), small-cell lung carcinoma (*n* = 4), non-small-cell lung cancer (*n* = 2), neuroendocrine tumor (*n* = 1), and mixed histology (*n* = 1). No significant association was found between pathology subtype and sex (χ^2^(5, *N* = 28) = 3.72, *p* = 0.59; and χ^2^(5, *N* = 28) = 3.09, *p* = 0.69, respectively). Given these findings, no evidence was found for an association between pathological subtype and either sex or smoking status in this sample.

### 3.8. Smoking Status

Data about smoking status were missing for four patients. Overall, cancer patients were more likely to be smokers (*n* = 243) than non-smokers (*n* = 193), χ^2^(*N* = 435) = 5.73, *p* = 0.02. Most patients smoked cigarettes only (*n* = 176), 57 patients smoked waterpipes only, and 10 patients smoked both cigarettes and waterpipes. On average, cigarette smokers consumed 1.27 packs per day, and waterpipe smokers consumed one waterpipe per day.

We examined differences in smoker versus non-smoker proportions by primary cancer site. Patients were more likely to be smokers than non-smokers for bladder cancer, (75.7% of bladder cancer patients were smokers vs. 53.9% of the remaining patients, χ^2^= 6.52, *p* = 0.011, OR = 2.66, 95% CI [1.23, 5.79]), lung cancer (75.9% vs. 54.3%, χ^2^ = 5.10, *p* = 0.024, OR = 2.65, 95% CI [1.11, 6.33]), and cervical cancer (Fisher’s exact text, *p* = 0.036). No OR was computed for cervical cancer in this case since all patients with cervical cancer in this sample were smokers.

Finally, the correlation between smoking status and sex by each cancer type revealed higher proportions of smokers among patients with bladder, lung, and laryngeal cancers in both males and females. For example, smoking prevalence exceeded 70% among bladder cancer patients and remained elevated in lung and laryngeal cancers. However, these differences did not reach statistical significance, likely due to limited sample sizes within subgroups. The percentages of patients by sex and smoking status per cancer types are presented in Table 4.

### 3.9. Barriers Along the Cancer Care Continuum Faced by the Patients

Barriers to cancer care were assessed across four domains: financial, system-level, geographic, and sociocultural. Valid percentages were used to account for missing responses (Table 5).

Overall, the most prevalent domain of barriers reported by patients in the sample was the financial domain (*n* = 427, 97.3%), followed very closely by the system-level domain (*n* = 419, 95.4%), indicating that nearly all patients encountered structural or cost-related challenges during care. Geographic barriers were also common (*n* = 317, 72.4%), while sociocultural barriers were reported by about half of the patients (*n* = 217, 49.7%).

Financial barriers were dominated by treatment-related costs and difficulties obtaining medications, highlighting affordability as a central challenge. Similarly, system-level barriers reflected both financial strain and access limitations, particularly in relation to medications and hospital services. Geographic barriers were primarily driven by transportation difficulties. Meanwhile, sociocultural barriers were less prevalent but pointed to gaps in emotional care as well as limited access to health information.

Among the examined sociodemographic variables (sex, age at presentation, area of residence, insurance status, and socioeconomic status), only SES was significantly associated with geographic barriers.

All patients classified as having very low SES (*n* = 23, 100%) reported at least one geographic barrier, compared to 75.7% of those with low SES (*n* = 190) and 63.4% of those with average SES (*n* = 104). This association was statistically significant (χ^2^ = 16.75, *p* < 0.001), with a small-to-moderate effect size (φ = 0.196).

## 4. Discussion

### 4.1. Main Findings of This Study

This study provides one of the first descriptive assessments of cancer patterns among socioeconomically vulnerable patients in northern Lebanon. Four key findings emerged: elevated rates of EOCRC, a high breast cancer burden among women, smoking-related malignancies, and major systemic barriers to care. Due to the non-population-based nature of the sample, ASPs were calculated instead of ASRs. Together, these findings suggest that behavioral risks, limited screening, and health system constraints may contribute to the observed cancer patterns. These findings highlight the potential value of integrating SES and geographic data into Lebanon’s cancer surveillance.

### 4.2. What Is Already Known on This Topic

Existing research shows that Lebanon, classified as an LMIC, has one of the highest cancer incidence rates in the Arab world [4]. Since 2019, the country’s healthcare sector has been severely weakened by multiple crises, including economic collapse and political instability, which may have affected timely access to cancer treatment [28]. Studies indicate that over 50% of cancers in Lebanon are diagnosed at advanced stages, particularly in socioeconomically disadvantaged areas like Tripoli, where national healthcare efforts have proven insufficient [10]. Additionally, access to screening programs, such as mammograms for breast cancer, has been particularly limited for low-income individuals [29]. Early detection is associated with improved prognoses and significantly higher survival rates [30].

### 4.3. EOCRC

Nearly 37% of CRC cases were diagnosed before age 50, with a mean age about ten years younger than national averages for both sexes [22]. This mirrors a global increase in EOCRC reported across high- and middle-income countries [31,32,33,34,35]. The reasons are multifactorial, involving diet, obesity, antibiotic exposure, and lifestyle factors [4,36]. In Lebanon, national screening guidelines recommend stool-based screening beginning at age 50 [22]; however, our results suggest that a sizable proportion of cases occur well before this threshold. In 2025, the incidence of colorectal cancer (CRC) in Lebanon is projected to rise to 28.8 per 100,000 males and 26.1 per 100,000 females, up from 23.2 and 20.2 per 100,000 in 2016, respectively [37]. Lebanon ranks second in the South-West Asia and North Africa (SWANA) region for CRC incidence, where it is also the fifth-most prevalent cancer [38]. Between 2005 and 2016, 10,284 new CRC cases were recorded, with 77.4% occurring in individuals aged 50 years and above (1), and over 80% of diagnoses reported at advanced stages (T3 and T4) [39]. Despite these statistics, CRC screening uptake remains low, contributing to late-stage detection. Public awareness remains low: 59% of Lebanese are unfamiliar with CRC, 83% are unaware of its risk factors, and 67% cannot identify its warning signs [40,41]. Furthermore, CRC is not prioritized within the Ministry of Public Health (MoPH) agenda, as evidenced by the early discontinuation of the National CRC campaign launched in 2019 [22]. Consequently, treatment costs escalated to USD 64,805 per patient, with 67% of the MoPH oncology drug budget from 2008 to 2013 allocated to CRC [41,42]. Despite strong evidence supporting the effectiveness of screening programs in reducing CRC incidence and mortality, such initiatives remain inactive, and screening uptake in Lebanon continues to be very low [42]. CRC ranked as the second-most prevalent cancer type in both sexes in our sample, and this high rate can be linked to modifiable behaviors such as lack of screening awareness, unhealthy lifestyle choices, and insufficient early detection [37]. Other risk factors should also be considered in future studies and larger endeavors that take into account biological and environmental contributors [36].

These findings support consideration of earlier screening strategies—around age 45—in high-risk underserved populations, which goes in the same direction as other studies in Lebanon [43]. Earlier symptom-based referral pathways and public awareness of EOCRC warning signs could also reduce diagnostic delays, especially as EOCRC often presents symptomatically [44,45].

### 4.4. High Burden of Breast Cancer

Breast cancer accounted for almost half of all female cancers in this cohort, exceeding national estimates (~39%) [13,46]. This pattern is consistent with projections showing breast cancer as the leading and fastest-growing malignancy in Lebanon [5,17,47]. The concentration of cases in vulnerable communities may reflect under-screening and late presentation. Previous studies have shown that many breast cancer cases are diagnosed at stage IV despite national screening campaigns [10], and have documented significant regional disparities in mammography uptake, largely attributable to financial, cultural, and informational barriers [11,48]. These regional differences and screening gaps are well documented [46,49]. Furthermore, breast cancer in this region tends to take a more aggressive course, possibly influenced by genetic factors reported in the region [50]. Addressing these barriers requires culturally sensitive outreach—particularly in northern Lebanon—through community health workers, faith-based networks, navigation services, and mobile screening units [51,52]. Interventions must also address gender norms and stigma, key factors influencing women’s willingness to seek early detection [53,54]. Lastly, reporting the stage at diagnosis and the molecular subtype of breast cancer [55], along with SES and residence data, can help track disparities in breast cancer presentation and outcomes across Lebanon.

### 4.5. Smoking-Related Cancers

The smoking-related cancer pattern—bladder cancer in men and high lung cancer rates in women—reflects Lebanon’s distinctive tobacco-use profile. The country has among the world’s highest rates of both cigarette and waterpipe smoking [56,57,58,59], and this trend is alarmingly seen in adolescents as well [60]. Waterpipe use, in particular, is now recognized as a significant risk factor for bladder cancer [25,58] because of prolonged exposure to carcinogenic aromatic amines and polycyclic hydrocarbons [61,62]. In males, bladder cancer is statistically tied with prostate cancer as the most common malignancy. This elevated incidence of bladder cancer may be partially attributed to excess smoking among the Lebanese population, as well as certain genetic factors that could be specific to the residents of the studied area [63,64]. The high proportion of smokers underscores the need for locally tailored tobacco-control measures—such as regulation of waterpipe cafés, producer taxation, and targeted cessation programs. Smoking is strongly linked to low SES, warranting interventions that address its social determinants [65]. Integrating cessation counseling within primary and oncology care can yield measurable health benefits even after diagnosis [66,67]. Finally, screening for bladder cancer in men of low SES who are smokers may warrant further investigation [63].

### 4.6. Sex Differences Across Non-Sex-Specific Cancer Types

Sex differences in cancer distribution in this cohort were limited and largely cancer-specific. While colorectal cancer showed a higher prevalence among males, consistent with the well-established pattern of males exhibiting higher incidence and mortality rates from colorectal cancer compared to females [68], and bladder cancer demonstrated a statistically significant association with male sex, in keeping with the widely reported finding that men have approximately four times the incidence rate of bladder cancer compared to women [69], other cancer types did not exhibit significant sex-based differences. These findings suggest that sex may play a role in certain malignancies but does not consistently influence cancer distribution across all tumor types in this population. Consistent with these findings, the multinomial logistic regression analysis did not identify sex as a significant overall predictor of cancer type distribution, supporting the absence of a consistent sex-based pattern across non-sex-specific malignancies.

### 4.7. Systemic Barriers to Cancer Care

This study demonstrated a multidimensional burden of barriers to cancer care in this community, with financial and system-level constraints affecting the majority of patients. Nearly all participants experienced at least one financial barrier, particularly difficulties affording treatment and obtaining medications, while geographic barriers—especially transportation—and sociocultural constraints were also common. These findings highlight how barriers co-occur and interact across the cancer care continuum, rather than acting as isolated factors. The predominance of financial toxicity is consistent with evidence from low- and middle-income countries (LMICs), where out-of-pocket expenditures frequently lead to delayed treatment, interruption of care, and poorer outcomes [70,71]. In Lebanon, the ongoing economic crisis has further exacerbated these challenges through drug shortages, reduced healthcare affordability, and disruptions in service delivery [9,72].

System-level barriers, including limited access to medications and hospitals, as well as communication challenges with healthcare providers, were also highly prevalent. These findings reflect structural weaknesses commonly reported in LMIC health systems, where fragmented care delivery, supply chain instability, and workforce limitations impede timely diagnosis and treatment [73]. In Lebanon, recent evidence highlights the collapse of pharmaceutical supply chains and strained hospital capacity as major contributors to delayed or interrupted oncology care [72,74]. Geographic barriers were likewise significant and disproportionately affected patients with lower socioeconomic status, consistent with literature showing that distance to care and transportation costs are major determinants of access in resource-limited settings [75]. Such disparities are particularly relevant in peripheral regions like North Lebanon, where specialized oncology services are less accessible.

Sociocultural barriers, including lack of social and emotional support and limited access to health information, were less frequent but remain clinically important, as they influence health-seeking behavior, treatment adherence, and patient navigation. These findings align with prior research demonstrating the role of health literacy, stigma, and social support in shaping cancer outcomes in underserved populations [76]. Compared to high-income countries, where barriers are more often related to system navigation, the magnitude and overlap of financial, structural, and geographic constraints observed here reflect patterns typical of fragile and crisis-affected health systems. Addressing these disparities requires integrated interventions, including financial protection mechanisms, strengthened medication supply chains, decentralized oncology services, and community-based navigation and education programs. The strong association between socioeconomic status and geographic barriers further underscores the need for targeted, equity-focused strategies.

### 4.8. Incorporating SES and Geographic Data in the National Cancer Database

Beyond site-specific findings, the study identifies a major gap—the lack of SES and geographic data in Lebanon’s NCR. Lebanon’s NCR currently records age, sex, and diagnosis but lacks standardized variables for income, education, insurance coverage, or district of residence [3]. Our study highlights the substantial burden of cancer and the disparities in healthcare access experienced by vulnerable communities in Tripoli. This aligns with previous research that has identified significant discrepancies in cancer burdens within LMICs. For instance, in 2023, a study conducted by Stefan and Tang reported that most new cancer cases occur in LMICs, which exhibit poorer health outcomes compared to high-income countries [77]. Moreover, predictive statistical analyses suggest that in the upcoming 50 years, LMICs will contribute to the majority of the worldwide increase in cancer cases [70].

The absence of SES and geographic variables limits the ability to quantify inequities in cancer incidence, stage at diagnosis, or survival. Our approach—linking patient-level medical data with contextual SES information collected through a community-based organization—demonstrates a practical model for integrating these dimensions into routine assessment. Incorporating SES and residence variables, in addition to real-time data updates, into the NCR and hospital databases would enable policymakers to quantify inequities among Lebanese regions in cancer-related health metrics based on SES and residence, identify geographic clusters of vulnerability, develop targeted interventions such as screening and awareness campaigns, and monitor progress toward equity in cancer outcomes [78,79].

## 5. Limitations

This study has several limitations. First, the data originate from a single NGO in Tripoli and may not represent the national population nor capture the broader cancer landscape in North Lebanon. The aim was to explore potential patterns of cancer in a socioeconomically vulnerable sub-population in Tripoli. The results are limited to patients who sought assistance from Sanabel Nour, potentially excluding other at-risk populations. This introduces a significant selection bias, as the sample is heavily weighted toward individuals experiencing the most severe socioeconomic distress, potentially overestimating the prevalence of very low SES and the severity of financial barriers across the region. Second, some cancer subtypes had small sample sizes, reducing statistical power for subgroup analyses. Given the exploratory nature of the study and limited sample sizes across subgroups, multivariable analyses were performed selectively to avoid overfitting and unstable estimates. Third, the absence of a defined population at risk precluded the calculation of incidence or prevalence measures; therefore, standardized proportions were used to describe relative patterns. This limits comparability with national and international statistics. Alternative epidemiological measures, such as prevalence or incidence within the study population, were not calculated because the dataset is not population-based and lacks a defined denominator representing the underlying population at risk. As the sample consists of patients presenting to a single NGO, any prevalence estimate would be subject to substantial selection bias and would not reflect population-level patterns. Therefore, age-standardized rates were calculated using external population denominators (GLOBOCAN) to provide a standardized, albeit non-population-representative, comparison. Furthermore, comparisons with national registry data should be interpreted cautiously due to differences in methodology, population structure, and time frame. Patients may have presented at different stages of their disease trajectory, introducing potential referral and survivorship bias. Additionally, incomplete data on stage at diagnosis and treatment outcomes precluded survival analyses. Finally, the dataset did not include distance from healthcare facilities. Despite these limitations, this study provides valuable regional insights into cancer patterns among disadvantaged populations and offers a feasible model for integrating equity data into cancer surveillance.

## 6. Conclusions

This study provides a focused assessment of cancer patterns among socioeconomically vulnerable patients in Tripoli, Lebanon. The findings highlight a notable burden of EOCRC, a high proportion of breast cancer, and an elevated prevalence of smoking-related malignancies, and widespread financial and system-level barriers to care. Importantly, these results reflect a highly selected population and should not be interpreted as representative of the general population. Together, these findings support the integration of socioeconomic, geographic, and barrier variables into national cancer surveillance systems to enable more accurate identification of vulnerable populations. While exploratory in nature, this study provides a practical model for incorporating equity-focused data into cancer research in low- and middle-income settings. Future studies with larger, population-based samples are needed to validate these findings and inform targeted interventions aimed at reducing disparities in cancer care and outcomes.

## Figures and Tables

**Table 1:** Cancer by primary site, sorted by decreasing order of frequency and broken down by sex. The rank in parentheses indicates the five most common types of cancer for each sex. Any cancer at a primary site with five or fewer cases was put into the “other” category (*n* = 440).

	By Sex Number of Cases (Rank)		Total		
Primary Site	Male	Female	Frequency	Percent	ASP (Rank)
Breast	1	140 (1st)	141	32	5.05 (1st)
Colorectal	21 (2nd)	22 (2nd)	43	9.8	0.78 (2nd)
Bladder	26 (1st)	11 (5th–6th)	37	8.4	0.73 (3rd)
Lymphoma	13 (4th)	20 (3rd)	33	7.5	0.56 (6th)
Lung	12 (5th)	18 (4th)	31	7.0	0.59 (5th)
Leukemia	7	9	16	3.6	0.26 (9th)
Prostate	15 (3rd)	-	15	3.4	0.68 (4th)
Larynx	7	5	12	2.7	0.22 (10th)
Uterus	-	11 (5th–6th)	11	2.5	0.44 (7th)
Stomach	8	1	9	2.0	0.17 (11th–12th)
Ovary	-	9	9	2.0	0.35 (8th)
Multiple Myeloma	6	3	9	2.0	0.17 (11th–12th)
Kidney	6	2	8	1.8	0.14 (13th–14th)
Brain and CNS	3	5	8	1.8	0.14 (13th–14th)
Connective and Soft Tissue	4	3	7	1.6	0.12 (15th–16th)
Thyroid	1	6	7	1.6	0.12 (15th–16th)
Cervix	-	6	6	1.4	0.11 (17th)
Other Sites	22	16	38	8.6	
Total	152	288	440	100	7.99

**Table 2:** Age at diagnosis by type and sex.

		Average	SD	Median	Min	Max
Breast	Female (*n* = 140) *	49.88	11.47	48.57	27.10	82.72
CRC	Female (*n* = 22)	55.02	14.40	57.29	30.67	75.52
	Male (*n* =21)	54.51	12.94	53.41	29.29	74.88
	Total (*n* = 43)	54.55	13.63	56.05	29.29	75.52
Bladder	Female (*n* = 11)	63.59	10.63	62.45	43.39	79.29
	Male (*n* = 26)	59.50	9.82	61.75	40.73	76.96
	Total (*n* = 37)	60.71	10.10	61.15	40.73	79.29
Lymphoma	Female (*n* = 20)	42.60	15.80	41.95	18.49	71.10
	Male (*n* = 13)	38.16	17.36	35.00	13.90	75.86
	Total (*n* = 33)	40.85	16.31	40.20	13.90	75.86
Lung	Female (*n* = 19)	61.62	7.48	63.38	49.77	73.46
	Male (*n* = 12)	61.61	10.37	60.96	46.02	79.75
	Total (*n* = 31)	61.61	8.54	61.58	46.02	79.75
Leukemia	Total (*n* = 16) ^†^	21.34	18.21	15.96	1.28	56.47
Prostate	Male (*n* = 15)	67.36	8.65	68.68	51.19	78.80
Any Type	*N* = 440	50.72	15.93	51.95	0.54	83.57

* One male with breast carcinoma was excluded from this table. ^†^ Stratification of data by sex was not performed for certain cancer types due to small group sizes (10 or fewer per group).

**Table 3:** Comparison of the sample mean age of CRC diagnosis to the national mean age, stratified by sex.

	M ± SD	National Mean Age	t-Value	*p*-Value	Cohen’s d
Male (*n* = 21)	54.51 ± 12.94	66	−4.068	<0.001	0.85
Female (*n* = 22)	55.02 ± 14.40	65	−3.250	<0.05	0.67

**Table 4:** Count and percentage of patients by sex, smoking status, and cancer type.

Primary Site	Sex	Non-Smoker	Smoker
CRC	Male	11 (52.4%)	10 (47.6%)
	Female	12 (60.0%)	8 (40.0%)
Bladder	Male	6 (23.1%)	20 (76.9%)
	Female	3 (27.3%)	8 (72.7%)
Lymphoma	Male	7 (53.8%)	6 (46.2%)
	Female	11 (55.0%)	9 (45.0%)
Lung	Male	5 (41.7%)	7 (58.3%)
	Female	2 (11.8%)	15 (88.2%)
Leukemia	Male	3 (42.9%)	4 (57.1%)
	Female	7 (77.8%)	2 (22.2%)
Larynx	Male	2 (28.6%)	5 (71.4%)
	Female	2 (40.0%)	3 (60.0%)
Stomach	Male	2 (25.0%)	6 (75.0%)
	Female	0 (0.0%)	1 (100.0%)
Multiple Myeloma	Male	3 (50.0%)	3 (50.0%)
	Female	1 (33.3%)	2 (66.7%)
Kidney	Male	4 (66.7%)	2 (33.3%)
	Female	0 (0.0%)	2 (100.0%)
Brain and CNS	Male	3 (100.0%)	0 (0.0%)
	Female	3 (60.0%)	2 (40.0%)
Connective and Soft Tissue	Male	2 (50.0%)	2 (50.0%)
	Female	1 (33.3%)	2 (66.7%)
Thyroid	Male	1 (0.0%)	1 (100.0%)
	Female	3 (50.0%)	3 (50.0%)

**Table 5:** Count and percentage of patient-reported barriers to receiving cancer-related healthcare.

Financial Domain	Obtaining Medication	373	85.4%
	Cost of Treatment	362	82.5%
	Follow-Up Tests Not Performed on Time	109	24.8%
	Skipping Medical Appointments	82	18.7%
	**Any Financial Domain Barrier**	**427**	**97.3%**
System-Level Domain	Cost of Treatment	362	82.5%
	Access to Medication	187	42.9%
	Access to Hospital	115	26.2%
	Follow-Up Tests Not Performed on Time	109	24.8%
	Skipping Medical Appointments	82	18.7%
	Communicating with Physician	33	7.5%
	**Any System-Level Domain Barrier**	**419**	**95.4%**
Geographic Domain	Transportation to Medical Appointments	233	53.2%
	Access to Hospital	115	26.2%
	Follow-Up Tests Not Performed on Time	109	24.8%
	Skipping Medical Appointments	82	18.7%
	**Any Geographic Domain Barrier**	**317**	**72.4%**
Sociocultural Domain	Follow-Up Tests Not Performed on Time	109	24.8%
	Lack of Emotional Support	99	22.6%
	Skipping Medical Appointments	82	18.7%
	Lack of Social Support	48	11.0%
	Getting Info about the Disease/Self-Care	16	3.7%
	**Any Sociocultural Domain Barrier**	**217**	**49.7%**

## Data Availability

The original contributions presented in this study are included in the article/Supplementary Material. Further inquiries can be directed to the corresponding authors.

## Supplementary Materials

The following supporting information can be downloaded at: https://www.mdpi.com/article/10.3390/diseases14050170/s1, Table S1: Case Processing Summary of the Multinomial Logistic Regression Model; Table S2: Model-fitting information of the model; Table S3: Pseudo R-square values for the model; Table S4: Parameter estimates for the model; Table S5: Mean, median age at diagnosis for each type of cancer, and count and percentage of patients diagnosed at ages 50 and above and 60 and above.

## Abbreviations

CI	Confidence Interval
CRC	Colorectal Cancer
EOCRC	Early-Onset Colorectal Cancer
ICD	International Classification of Diseases
IRB	Institutional Review Board
LMICs	Low- and Middle-Income Countries
M/F	Male-to-Female ratio
MoPH	Ministry of Public Health
NCR	National Cancer Registry
NGO	Non-Governmental Organization
SD	Standard Deviation
SES	Socioeconomic Status
SES-C	Socioeconomic Status Composite Scale
T3/T4	Tumor Stages 3 and 4
USD	United States Dollar
χ^2^	Chi-Square
