# Supplementary material for: Cancer Patterns and Barriers to Care Among Socioeconomically Vulnerable Populations in Tripoli: A Descriptive Study from a Local NGO
Source: Diseases. 2026 May 12;14(5):170. doi: 10.3390/diseases14050170 (PMC13206300; doi:10.3390/diseases14050170)
Supplement: Supplementary file 1 [file diseases-14-00170-s001.zip › diseases-4219523-supplementary.pdf]

# Supplementary Material

## 1) Multinomial Logistic Regression Output:

| Case Processing Summary | | *N* | Marginal Percentage |
|---|---|---|---|
| **Primary Site** | Colorectal | 43 | 25.1% |
| | Bladder | 37 | 21.6% |
| | Lymphoma | 33 | 19.3% |
| | Lung | 30 | 17.5% |
| | Leukemia | 16 | 9.4% |
| | Larynx | 12 | 7.0% |
| **Sex** | Male | 86 | 50.3% |
| | Female | 85 | 49.7% |
| **Valid** | | 171 | 100.0% |
| **Missing** | | 0 | |
| **Total** | | 171 | |
| **Subpopulation** | | 2 | |

Table S1: Case Processing Summary of the Multinomial Logistic Regression Model

| Model | Model Fitting Criteria | Likelihood Ratio Tests | | |
|---|---|---|---|---|
| | -2 Log Likelihood | Chi-Square | df | Sig. |
| **Intercept Only** | 49.652 | | | |
| **Final** | 40.085 | 9.567 | 5 | 0.088 |

Table S2: Model-fitting information of the model

| Pseudo $R^2$ | Value |
|---|---|
| Cox and Snell | 0.054 |
| Nagelkerke | 0.056 |
| McFadden | 0.016 |

Table S3: Pseudo R-square values for the model

**Parameter Estimates:**

| Primary Site* | | B | Std. Error | Wald | df | Sig. | Exp(B) | 95% CI for Exp(B) | |
|---|---|---|---|---|---|---|---|---|---|
| | | | | | | | | Lower Bound | Upper Bound |
| **Bladder** | Intercept | -0.693 | 0.369 | 3.523 | 1 | 0.061 | | | |
| | [Sex=1] | 0.907 | 0.472 | 3.696 | 1 | 0.055 | 2.476 | 0.982 | 6.241 |
| | [Sex=2] | 0** | . | . | 0 | . | . | . | . |
| **Lymphoma** | Intercept | -0.095 | 0.309 | 0.095 | 1 | 0.758 | | | |
| | [Sex=1] | -0.384 | 0.469 | 0.671 | 1 | 0.413 | 0.681 | 0.272 | 1.708 |
| | [Sex=2] | 0** | . | . | 0 | . | . | . | . |
| **Lung** | Intercept | -0.201 | 0.318 | 0.399 | 1 | 0.528 | | | |
| | [Sex=1] | -0.359 | 0.482 | 0.555 | 1 | 0.456 | 0.698 | 0.272 | 1.795 |
| | [Sex=2] | 0** | . | . | 0 | . | . | . | . |
| **Leukemia** | Intercept | -0.894 | 0.396 | 5.103 | 1 | 0.024 | | | |
| | [Sex=1] | -0.205 | 0.589 | 0.121 | 1 | 0.728 | 0.815 | 0.257 | 2.585 |
| | [Sex=2] | 0** | . | . | 0 | . | . | . | . |
| **Larynx** | Intercept | -1.482 | 0.495 | 8.943 | 1 | 0.003 | | | |
| | [Sex=1] | 0.383 | 0.660 | 0.336 | 1 | 0.562 | 1.467 | 0.402 | 5.350 |
| | [Sex=2] | 0** | . | . | 0 | . | . | . | . |

*The reference category is Colorectal Cancer

**This parameter is set to zero because it is redundant.

Table S4: Parameter estimates for the model

**2) Age distribution metric for each type of cancer**:

| **Primary Site** | **Age at first diagnosis** | | **Count ≥ 50 years** | **% ≥ 50 years** | **Count ≥ 60 years** | **% ≥ 60 years** |
|---|---|---|---|---|---|---|
| | Mean | Median | | | | |
| Breast | 49.82 | 48.52 | 62 | 44% | 31 | 22% |
| Colorectal | 54.77 | 56.05 | 27 | 62.8% | 18 | 41.9% |
| Bladder | 60.71 | 62.15 | 28 | 75.7% | 21 | 56.8% |
| Lymphoma | 40.85 | 40.2 | 10 | 30.3% | 5 | 15.2% |
| Lung | 61.22 | 61.46 | 28 | 93.3% | 17 | 56.7% |
| Leukemia | 21.34 | 15.96 | 1 | 6.3% | 0 | 0% |
| Prostate | 67.36 | 68.68 | 15 | 100% | 12 | 80% |
| Larynx | 58.23 | 58.66 | 10 | 83.3% | 5 | 41.7% |
| Uterus | 56.48 | 59.89 | 8 | 72.7% | 5 | 45.5% |
| Stomach | 55.99 | 62.48 | 6 | 66.7% | 6 | 66.7% |
| Ovary | 57.03 | 52.98 | 6 | 66.7% | 4 | 44.4% |
| Multiple Myeloma | 58.72 | 54.44 | 7 | 77.8% | 3 | 33.3% |
| Kidney | 49.89 | 49.99 | 4 | 50% | 1 | 12.5% |
| Brain & CNS | 37.63 | 42.91 | 2 | 25% | 2 | 25% |
| Connective & Soft Tissue | 40.98 | 42.99 | 3 | 42.9% | 1 | 14.3% |
| Thyroid | 42.57 | 39.32 | 2 | 28.6% | 1 | 14.3% |
| Cervix | 55.53 | 53.82 | 4 | 66.7% | 1 | 16.7% |
| Other | 42.77 | 46.64 | 17 | 43.6% | 6 | 15.4% |

Table S5: Mean, median age at diagnosis for each type of cancer, and count and percentage of patients diagnosed at ages 50 and above and 60 and above.

### 3) Data collection form

| 1. Patient Information and SES |
|---|

- Patient code: _________________
- Sex: □ M □ F
- Residence: __________________
- Phone Number: ____________________
- Date of Birth: _______________
- Age (years): _____
- Date of interview: _____________
- Nationality: _______________; Marital Status: _________; Education: ___________
- Interviewing: □ Patient □ Proxy (specify): ___________
- Health insurance: □ No □ Yes (specify): __________
- MoPH card? □ No □ Yes

| أسئلة **InCharge** | **1** | **10** | **الرقم** |
|---|---|---|---|
| 1. ما هو شعورك حول مستوى ضغطك المالي اليوم؟ | ضغط ساحق | لا ضغط على الإطلاق | |
| 2. ما مدى رضاك عن وضعك المالي الحالي؟ | غير را ض/ية على الإطلاق | را ض/ية | |
| 3. ما هو شعورك حول وضعك المالي الحالي؟ | أشعر بالعجز | أشعر بالراحة | |
| 4. كم مرة تقلق/ين بشأن قدرتك على تلبية نفقات المعيشة الشهرية العادية؟ | قلق طوال الوقت | لا قلق أبدا | |
| 5. ما مدى ثقتك في أنه يمكنك العثور على المال لدفع ثمن أي طارئ مالي يكلف حوالي 5 مليون ل.ل. | لا ثقة | ثقة عالية | |
| 6. كم مرة يحدث هذا لك: تريد/ين الخروج لتناول الطعام أو الذهاب إلى السينما أو القيام بنشاط آخر ولا تذهب/ين لأنك لا تستطيع/ين دفع الثمن؟ | طوال الوقت | أبداً | |
| 7. كم مرة تجد/ين نفسك تحاول/ين أن تتدبر/ي أمرك مادياً لتعيش/ي بانتظار الأجر المقبل؟ | طوال الوقت | أبداً | |
| 8. كيف هو شعورك بالضغط تجاه ماليّتك الشخصية بشكل عام؟ | ضغط ساحق | لا ضغط على الإطلاق | |
| | | | **المجموع** |

| أسئلة **SES-C** | **خيارات الإجابات** | | | |
|---|---|---|---|---|
| 1. إلى أي طبقة اجتماعية تنتمي؟ | الفقيرة (1) | الوسطى الدنيا (2) | الوسطى العليا (3) | الثرية (4) |
| 2. مقياس InCharge للضائقة/الرفاهية المالية | 8 – 25 points (1) | 26 – 50 points (2) | 51 – 75 points (3) | >75 points (4) |
| 3. الدخل الشهري للأسرة ($) | لا مدخول (1) | أقل من 150 (2) | 151 – 450 (3) | أكثر من 450 (4) |
| 4. هل أنت مديون؟ | كلا (1) | نعم (0) | | |
| 5. هل تحصل على مساعدة مالية من الآخرين؟ | كلا (2) | نعم، أحيانا (1) | نعم، بانتظام (0) | |
| 6. عدد الأشخاص في المنزل: _______ | عدد الغرف (دون الحمامات والمطابخ): ____ | | | القسمة: _____ |
| أكثر من 3 أشخاص/ غرفة (1)؛ 1.51 – 3 ش/غ (2)؛ 1.1 – 1.5 ش/غ (3)؛ 0.51 – 1 ش/غ (4)؛ 0 – 0.5 ش/غ (5) | | | | |
| 7. المستوى التعليمي للمريض | أمّي (1) | مدرسي (2) | جامعي (3) | |
| 8. المستوى التعليمي لرب العائلة | أمّي (1) | مدرسي (2) | جامعي (3) | |
| 9. وضع العمل للمريض | يعمل (1) | لا يعمل (0) | | |
| 10. وضع العمل لرب العائلة | يعمل (1) | لا يعمل (0) | **المجموع** | _________ |
| **الفئة الاجتماعية الاقتصادية** | ■ منخفضة جداً (6 – 10 نقطة) | ■ منخفضة (11 – 16 نقطة) | ■ متوسطة (17 – 22 نقطة) | ■ عالية (23- 28 نقطة) |

## 2. Physician Information

- Physician Name: ____________________

## 3. Tumor Registry Info

- Phase of the continuum at presentation:
  □ Dx → start of treatment; □ During treatment; □ Survivorship; □ Relapse; □ End of life
- Tumor Primary Site: __________________
- ICD category: ______________________; NCR entry: _____________________
- ICD code: _______________________
- Pathology: ______________________
- IHC: ______________________; Luminal subtype for BC: ______________
- Date of 1st diagnosis: ____/_______/____; Age at 1st diagnosis: __________

-Stage at 1st diagnosis: _______________________________ (Staging system: _____ )

-Stage at presentation: _______________________________________

- Metastasis: □ Yes □ No; Site (where to): ____________________

| ***Treatment Information*** | **Previous** | **Current** |
|---|---|---|
| **Received/ are receiving any cancer treatment?** | □ **Yes** □ **No** | □ **Yes** □ **No** |
| **If yes, what is the type of treatment?** | | |
| **Frequency of treatment** | | |
| **Duration of treatment** | | |

- **Chemotherapy:** Medications used: ____________________________________________
  **Radiotherapy:** Site(s) ___________________; Dosage: ________________
  **Other cancer medications:** ___________________

**-Have you had surgery related to cancer? □ Yes □ No**

**Surgery 1:** □ Yes; □ No;

**Surgery 1 type:** _________________; **Surgery 1 date:** ______________________

**Surgery 2:** □ Yes; □ No;

**Surgery 2 type:** _________________; **Surgery 2 date:** ______________________

**Did any swelling occur in your arm or leg after cancer surgery?** □ Yes □ No

## 4. Comorbidities

| Disease | ICD category | ICD code | Proof |
|---|---|---|---|
| | | | □ Yes □ No |
| | | | □ Yes □ No |
| | | | □ Yes □ No |
| | | | □ Yes □ No |
| | | | □ Yes □ No |

Previous history of malignancy: □ Yes □ No; If yes, specify type: ________________
Predisposing condition: □ Yes □ No; If yes, specify type: ________________

## 5. Family History

Number of the patient's family who have cancer: _______________________________

Number of 1st-degree relatives who have cancer: _______________________________

Number of 1st-degree relatives with the same type of cancer as the patient: ___________

| Relative | Degree of relation | Type of cancer | Same as patient | Age at diagnosis |
|---|---|---|---|---|
| | | | □ Yes □ No | |
| | | | □ Yes □ No | |
| | | | □ Yes □ No | |
| | | | □ Yes □ No | |
| | | | □ Yes □ No | |

## 6. Information

| | Diagnosis | | Prognosis | |
|---|---|---|---|---|
| **Patient** | **□ Yes** | **□ No** | **□ Yes** | **□ No** |
| **Family** | **□ Yes** | **□ No** | **□ Yes** | **□ No** |

## 7. Nutrition

- In the past 4 weeks, the patient's weight has: □ Decreased □ Not changed □ Increased
- How is the patient's appetite/ food intake? □ Decreased □ Not changed □ Increased

## 8. Lifestyle

- **Current Smoker** □ Yes □ No
- Type of smoking: □ Cigarettes □ Shisha
- How much: ____ Packs/day; ______ Shishas/day
- Duration of smoking: ____ years (Pack-year: ___)

## 9. Active Symptoms

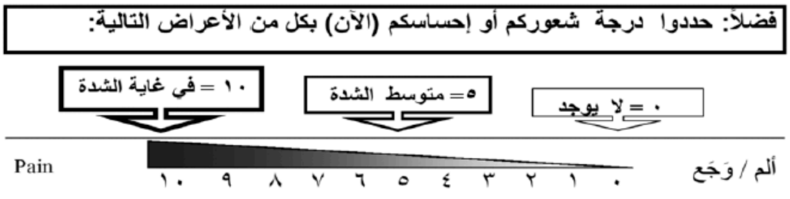


**If yes, where is your pain?** ______________________________

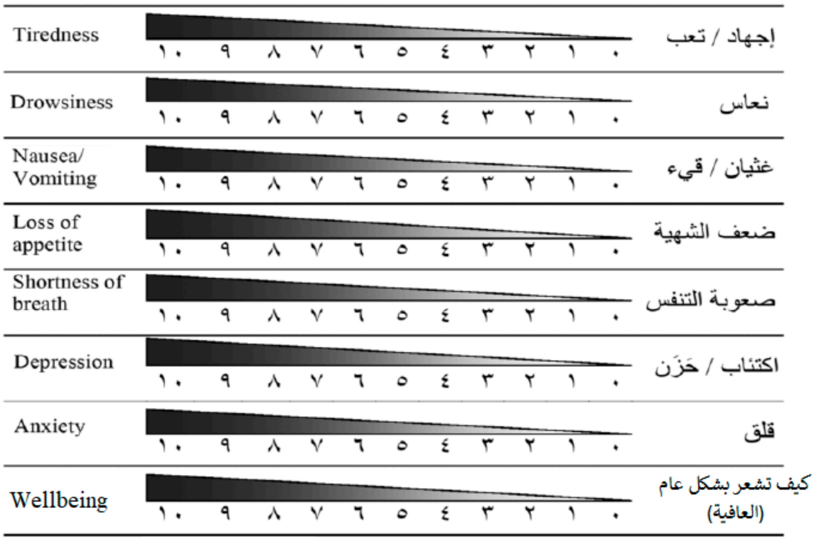


- **Total ESAS Score:** _______
- **Other symptoms:** ________________________________________________

## 10. Hospice Screen

- **Do you use a cane, walker, have difficulty walking?** □ Yes □ No
- **Do you need help caring for yourself?** □ Yes □ No
- **Do you do your work as normal before the onset of disease?** □ Yes □ No
- **Are you able to do housework and hobbies?** □ Yes □ No
- **Do you have memory problems?** □ Yes □ No

| Modified ADL | 1 | 2 | 3 | 4 |
|---|---|---|---|---|
| **Bathing** | Completely dependent (bed bath) | Needs personal assistance (in and out of tub) | Uses a device (shower stool) | Independent |
| **Dressing** | Completely dependent | Needs personal assistance (buttoning, choosing clothes) | Uses a device (i.e. reacher) | Independent |
| **Toileting** | Completely dependent | Needs personal assistance | Uses a device (walker, cane) | Independent |
| **Transfer** | Completely dependent | Needs personal assistance | Uses a device (walker, cane) | Independent |
| **Continence** | Completely dependent (catheter or diapers) | Needs personal assistance (to position urinal/bedpan) | Uses a device(urinal or bedpan) | Independent |
| **Feeding** | Completely dependent (feeding tube or not eating) | Needs personal assistance (fed by another) | Uses a device | Independent |
| **Score:** | __________ | ■ ≤ 18 | ■ > 18 | |

| PPS Level | Ambulation | Activity & Evidence of Disease | Self-Care | Intake | Conscious Level |
|---|---|---|---|---|---|
| 100% | Full | Normal activity & work<br>No evidence of disease | Full | Normal | Full |
| 90% | Full | Normal activity & work<br>Some evidence of disease | Full | Normal | Full |
| 80% | Full | Normal activity *with* Effort<br>Some evidence of disease | Full | Normal or reduced | Full |
| 70% | Reduced | Unable Normal Job/Work<br>Significant disease | Full | Normal or reduced | Full |
| 60% | Reduced | Unable hobby/house work<br>Significant disease | Occasional assistance necessary | Normal or reduced | Full<br>or Confusion |
| 50% | Mainly Sit/Lie | Unable to do any work<br>Extensive disease | Considerable assistance required | Normal or reduced | Full<br>or Confusion |
| 40% | Mainly in Bed | Unable to do most activity<br>Extensive disease | Mainly assistance | Normal or reduced | Full or Drowsy<br>+/- Confusion |
| 30% | Totally Bed Bound | Unable to do any activity<br>Extensive disease | Total Care | Normal or reduced | Full or Drowsy<br>+/- Confusion |
| 20% | Totally Bed Bound | Unable to do any activity<br>Extensive disease | Total Care | Minimal to sips | Full or Drowsy<br>+/- Confusion |
| 10% | Totally Bed Bound | Unable to do any activity<br>Extensive disease | Total Care | Mouth care only | Drowsy or Coma<br>+/- Confusion |
| 0% | Death | - | - | - | - |

| **Hospice Criteria:** | | |
|---|---|---|
| 1. | Cancer diagnosis is confirmed through pathology or radiology | □ Yes □ No |
| 2. | The patient is no longer receiving curative treatment | □ Yes □ No |
| 3. | There is evidence of end-stage disease, or metastasis, or prognosis ≤ 6 months | □ Yes □ No |
| 4. | Lab / diagnostic studies have been done recently to support disease progression | □ Yes □ No |
| 5. | PPS ≤ 60% or declining PPS | □ Yes □ No |
| 6. | Modified ADL score of ≤ 18 | □ Yes □ No |
| 7. | Evidence of active symptoms as per ESAS | □ Yes □ No |
| 8. | The patient and/or their family chose palliative care | □ Yes □ No |

**Patient needs hospice care?** □ Yes □ No

## 10. Barriers

| | **Has the problem?** | | |
|---|---|---|---|
| **Communication with physician problems** | □ Yes | □ No | □ NA |
| **Getting health information about the disease/ self-care** | □ Yes | □ No | □ NA |
| **Access to Medications** | □ Yes | □ No | □ NA |
| **Difficulty obtaining your medicines due to finances** | □ Yes | □ No | □ NA |
| **Access to Hospital** | □ Yes | □ No | □ NA |
| **Cost of Treatment/ Hospitalization** | □ Yes | □ No | □ NA |
| **Unable to take cancer medication/treatment on time** | □ Yes | □ No | □ NA |
| **Transportation difficulty to medical appointments** | □ Yes | □ No | □ NA |
| **Skipping Appointments** | □ Yes | □ No | □ NA |
| **Cancer-related follow-up tests not performed on time** | □ Yes | □ No | □ NA |
| **Lack of adequate social support** | □ Yes | □ No | □ NA |
| **Lack of emotional support** | □ Yes | □ No | □ NA |
